# Women Specific Characteristics and 1-Year Outcome Among Patients Hospitalized for Peripheral Artery Disease: A Monocentric Cohort Analysis in a Tertiary Center

**DOI:** 10.3389/fcvm.2022.824466

**Published:** 2022-02-07

**Authors:** Grégoire Détriché, Alexis Guédon, Nassim Mohamedi, Olfa Sellami, Charles Cheng, Alexandre Galloula, Guillaume Goudot, Lina Khider, Hélène Mortelette, Jonas Sitruk, Nicolas Gendron, Marc Sapoval, Pierre Julia, David M. Smadja, Tristan Mirault, Emmanuel Messas

**Affiliations:** ^1^Vascular Medicine Department, Hôpital Europeen Georges-Pompidou, Assistance Publique Hôpitaux de Paris, Université de Paris (APHP-CUP), Paris, France; ^2^Université de Paris, Innovative Therapies in Haemostasis, INSERM, Paris, France; ^3^Biosurgical Research Lab (Carpentier Foundation), Assistance Publique Hôpitaux de Paris, Centre-Université de Paris (APHP-CUP), Paris, France; ^4^Université de Paris, Paris Research Cardiovascular Center (PARCC), INSERM U970, Paris, France; ^5^Hematology Department, Hôpital Europeen Georges-Pompidou, Assistance Publique Hôpitaux de Paris, Université de Paris (APHP-CUP), Paris, France; ^6^Interventional Radiology Department, Hôpital Europeen Georges-Pompidou, Assistance Publique Hôpitaux de Paris, Universit de Paris (APHP-CUP), Paris, France; ^7^Vascular Surgery Department, Hôpital Europeen Georges-Pompidou, Assistance Publique Hôpitaux de Paris, Université de Paris (APHP-CUP), Paris, France

**Keywords:** peripheral arterial disease, cardiovascular disease, sex differences, vascular surgical procedure, lower extremity arterial disease

## Abstract

Although women have lower age-standardized cardiovascular disease incidence, prevalence, and death-related rates than men, there are also reports indicating that women with cardiovascular disease receive less care, fewer investigations, and have poorer outcomes after a coronary event. The aims of this study were to compare the characteristics of men and women hospitalized for peripheral artery disease (PAD), their cardiovascular and limb outcomes, and their 1-year mortality. The study is a prospective registry collecting data about all consecutive patients hospitalized for PAD within the vascular department of the tertiary center Georges-Pompidou European Hospital (Paris, France). Patients were required to have one of three inclusion criteria: previous revascularization of the lower limb or any lower limb artery occlusion due to an atherosclerotic vascular disease or hemodynamic evidence of PAD. Exclusion criteria were patients with lower extremity arterial occlusion due to another cause. All patients were followed-up for at least 12 months after the initial hospitalization. Among the 235 patients included, there were 61 women (26%), older than men with a median age of 75.6 and 68.3 years, respectively. Main cardiovascular risk factors and comorbidities were similar for men and women except more former or current smokers [145 (83.4%) vs. 33 (54.1%)] and more history of coronary heart disease [42 (24.1%) vs. 7 (11.5%)] in men. Most patients [138 (58.8%)] had critical limb ischemia and 97 (41.3%) had claudication, with no difference for sex. After discharge, 218 patients received an antithrombotic therapy (93.2%), 195 a lipid-lowering drug (83.3%), 185 an angiotensin converting enzyme inhibitor or angiotensin-receptor blocker (78.9%), similarly between sex. At 1-year, overall mortality, major adverse cardiovascular events, major adverse limb events did not differ with 23 (13.2%), 11 (6.3%) and 32 (18.4%) in men, and 8 (13.1%), 3 (4.9%), 15 (24.6%) in women, respectively, despite the difference in age. Overall mortality, cardiovascular outcomes, limb revascularization or amputation did not differ between men and women, 1-year after hospitalization for PAD although the latter were older, less smoker and had less coronary artery disease. Due to the small size of this cohort, larger studies and future research are needed to better understand sex-specific mechanisms in the pathophysiology and natural history of PAD.

## Introduction

“Despite being responsible for causing 35% of deaths in women each year, cardiovascular disease in women remains understudied, under-recognized, and under-treated, with women under-represented in clinical trials” stated The Lancet women and cardiovascular disease Commission in May 2021 (1). Although women have lower age-standardized cardiovascular disease incidence, prevalence, and death rates than men (2) there are also reports indicating that women with cardiovascular disease receive less care, fewer investigations, and have poorer outcomes after a coronary event (3–6). The lower extremity arterial disease, also known as peripheral artery disease (PAD) in some extent, is the manifestation of the atherosclerotic cardiovascular disease (ASCVD) at the lower limbs level (7). Most patients presenting with asymptomatic PAD do not have a clinical history of cardiac or cerebral ischemic events, although they are at high risk for stroke, myocardial infarction and cardiovascular death (8, 9) with a 10-year cardiovascular mortality of 18.7% in men and 12.6% in women with a low ankle-brachial index (≤ 0.90) (10). Sex difference in symptomatic PAD has been scantly studied and women may be less likely to undergo revascularization than men and more likely under-treated (1, 6, 11–13).

The aims of this study were to compare the characteristics of patients hospitalized for PAD, their cardiovascular and limb outcomes, and their mortality at 1 year according to sex.

## Materials and Methods

### Study Design

The study is a prospective monocentric registry collecting exhaustive data about all patients consecutively hospitalized for PAD within the Georges-Pompidou European Hospital vascular department (APHP-Université de Paris). The methodology of the registry has already been published elsewhere (14). The protocol was approved by the local ethics committee (IRB CPP Sud Ouest et outre mer II 07 021 08) and patients provided written informed consent before enrollment. The data were retrieved from the patient's computerized record. We collected demographic, clinical, laboratory and imaging data as well as all the complications that had occurred for each patient followed.

### Study Population

Eligible patients were at least 18 years of age and hospitalized for symptomatic lower extremity artery disease. Patients were required to have one of three inclusion criteria: previous revascularization of the lower limb or any lower limb artery occlusion due to an ASCVD or hemodynamic evidence of PAD as evidenced by an ankle-brachial index (ABI) of 0.90 or less or a toe-brachial index (TBI) of 0.60 or less, in accordance with current guidelines (7, 15). Exclusion criteria were patients with lower extremity arterial occlusion due to another cause than ASCVD.

All patients were followed-up for at least 12 months after the initial hospitalization. Patient care was provided according to the usual practice, without any change in management strategy. Phone contacts with the patients or their physicians have been performed when required. The primary end point was all cause mortality within 1 year. Registrar's offices have been consulted when required. Secondary end points were the occurrence of any event in the composite of cardiovascular death, myocardial infarction, or ischemic stroke defined as major adverse cardiovascular event (MACE), the occurrence of any event in the composite of lower limb major amputation or revascularization defined as major adverse limb event (MALE), or the occurrence of cancer.

### Statistical Analysis

Discrete variables are presented as number and percentage, and continuous variables as median and interquartile range (IQR, 25th−75th percentile). Comparisons were made using chi-square test (or Fisher exact tests, when appropriate) for discrete variables, and Mann-Whitney test for continuous variables. All subsequent *p*-values are reported for 2-tailed tests with a 5% threshold. Overall survival, MACE and MALE were calculated using the Kaplan-Meier method, and the values were compared using the log-rank test. All analyses were performed using SPSS software vs. 13.0 (SPSS Inc., Chicago, IL) and GraphPad Prism 5 (GraphPad Software, Inc., La Jolla, CA, USA).

## Results

### Cohort and Baseline Characteristics

From January 2018 to January 2019 a total of 235 patients were included. Table 1 shows the characteristics of the patients. The median age was 70.0 (59.3–79.8) years. There were 61 (26%) women. They were older than men, with a median age of 75.6 (60.4–82.6) and 68.3 (59.3–78.6) years, respectively *(p* = 0.04). Main cardiovascular risk factors were similar in both sex, with a trend for more hypertension in women (78.7%, *p* = 0.15) and diabetes mellitus in men (41.9%, *p* = 0.70). However, tobacco use was significantly different with more former or current smokers in men (*p* < 0.001). In parallel, a history of coronary heart disease, and myocardial infarction in particular, was significantly higher in men [42 men (24.1%) vs. 7 women (11.5%), *p* = 0.04]. Other comorbidities reflected the clinical complexity of the patients with 12 (5.1%) patients with heart failure, 29 (12.3%) with stroke or transient ischemic attack, and 25 (10.7%) with chronic obstructive pulmonary disease. The majority of patients, 138 (58.8%) had critical limb ischemia defined as pain of the lower limb while at rest or tissue loss and 97 (41.3%) had claudication. Neither the PAD Rutherford category nor previous revascularizations differed between sex. Patients already had a history of vascular revascularization of the lower limbs with bypass for 41 (17.4%) patients and endovascular procedure for 81 (34.5%) patients. Already 29 (12.3%) patients had previous amputation. Half of the patients has undergone a revascularization before discharge. After discharge, 218 patients received an antithrombotic therapy (93.2%), 195 a lipid-lowering drug (83.3%), 185 an angiotensin converting enzyme inhibitor or angiotensin-receptor blocker (78.9%), similarly between sex.

**Table 1:** Baseline characteristics and sex comparison.

	**Total (*n* = 235)**	**Men (*n* = 174)**	**Women (*n* = 61)**	***p*-value**
Median age - years (IQR)	70.0 (59.3–79.8)	68.6 (59.3–78.6)	75.6 (60.4–82.6)	**0.04**
Hypertension	168 (71.5)	120 (69.0)	48 (78.7)	0.15
Hyperlipemia	149 (63.4)	110 (63.2)	39 (63.9)	0.92
Smoking status				**<0.001**
Never smoked	57 (24.3)	29 (16.7)	28 (45.9)	
Current smoker	68 (28.9)	53 (30.5)	15 (24.6)	
Former smoker	110 (46.8)	92 (52.9)	18 (29.5)	
Diabete mellitus	94 (40.0)	73 (41.9)	21 (34.4)	0.70
Stroke	20 (8.5)	12 (6.9)	8 (13.1)	0.13
Transient ischemic attack	9 (3.8)	5 (2.9)	4 (6.6)	0.24
Coronary artery disease	57 (24.3)	48 (27.6)	9 (14.8)	**0.04**
Myocardial infarction	49 (20.9)	42 (24.1)	7 (11.5)	**0.04**
Heart failure	12 (5.1)	9 (5.2)	3 (4.9)	1.00
Chronic obstructive pulmonary disease	25 (10.7)	17 (9.8)	8 (13.3)	0.45
Abdominal aortic aneurysm	14 (6.0)	12 (6.9)	2 (3.3)	0.53
Peripheral artery disease Rutherford category				0.11
Category 1/2/3: Intermittent claudication	97 (41.3)	76 (43.7)	21 (34.4)	
Category 4: Pain while at rest	6 (2.6)	6 (3.4)	0 (0.0)	
Category 5: Minor trophic lesion	126 (53.6)	88 (50.6)	38 (62.3)	
Category 6: Major trophic lesion	6 (2.5)	4 (2.3)	2 (3.3)	
Previous bypass revascularization	41 (17.4)	32 (18.4)	9 (14.8)	0.52
Previous endovascular revascularization	81 (34.5)	65 (37.4)	16 (26.2)	0.12
Previous cardiovascular rehabitilation	20 (8.7)	14 (8.2)	6 (10.2)	0.65
Previous lower limb amputation	29 (12.3)	26 (14.9)	3 (4.9)	
Major amputation	5 (2.1)	5 (2.9)	0	0.33
Minor amputation	24 (10.2)	21 (12.1)	3 (4.9)	0.11
Revascularization before discharge	132 (56.2)	93 (53.4)	39 (63.9)	0.16
Treatment after discharge				
Anticoagulant	35 (14.9)	26 (14.9)	9 (14.8)	0.97
Aspirin	177 (75.3)	129 (74.1)	48 (78.7)	0.48
Clopidogrel	77 (32.8)	58 (33.3)	19 (31.1)	0.75
Dual antiplatelet therapy	49 (20.9)	35 (20.1)	14 (23.0)	0.87
ACE inhibitor	133 (56.6)	101 (58.0)	32 (52.5)	0.45
Angiotensin II receptor antagonist	52 (22.1)	40 (23.0)	12 (19.7)	0.59
Statin	194 (82.6)	145 (83.3)	49 (80.3)	0.59
Ezetimibe	10 (4.3)	10 (5.7)	0	0.07

*IQR, interquartile range (25th – 75th percentile); ACE, angiotensin converting enzyme. Significant p values are in bold*.

### One-Year Outcomes

**Figure 1:**
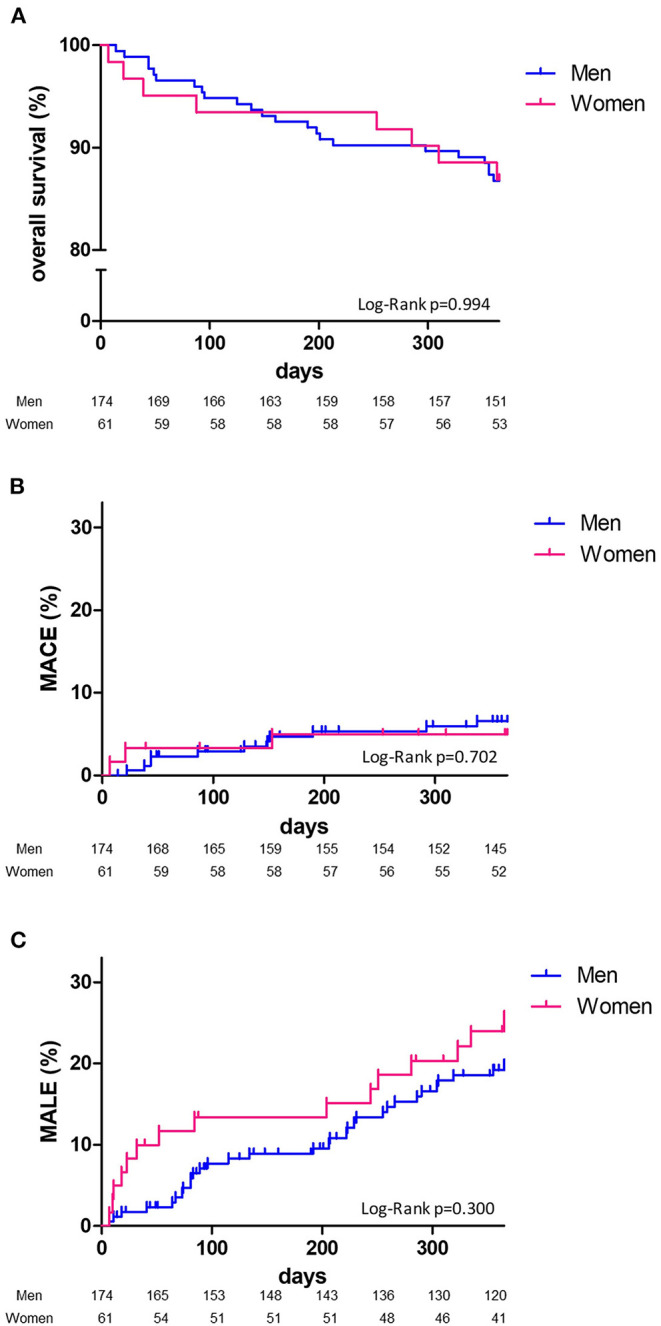
One-year Kaplan-Meier survival curves. Overall survival **(A)**, major adverse cardiovascular events: MACE **(B)**, major adverse limb events: MALE **(C)** and comparison according to sex (men, blue line; women, red line) with Log Rank test comparisons between men (blue) and women (red).

**Figure 2:**
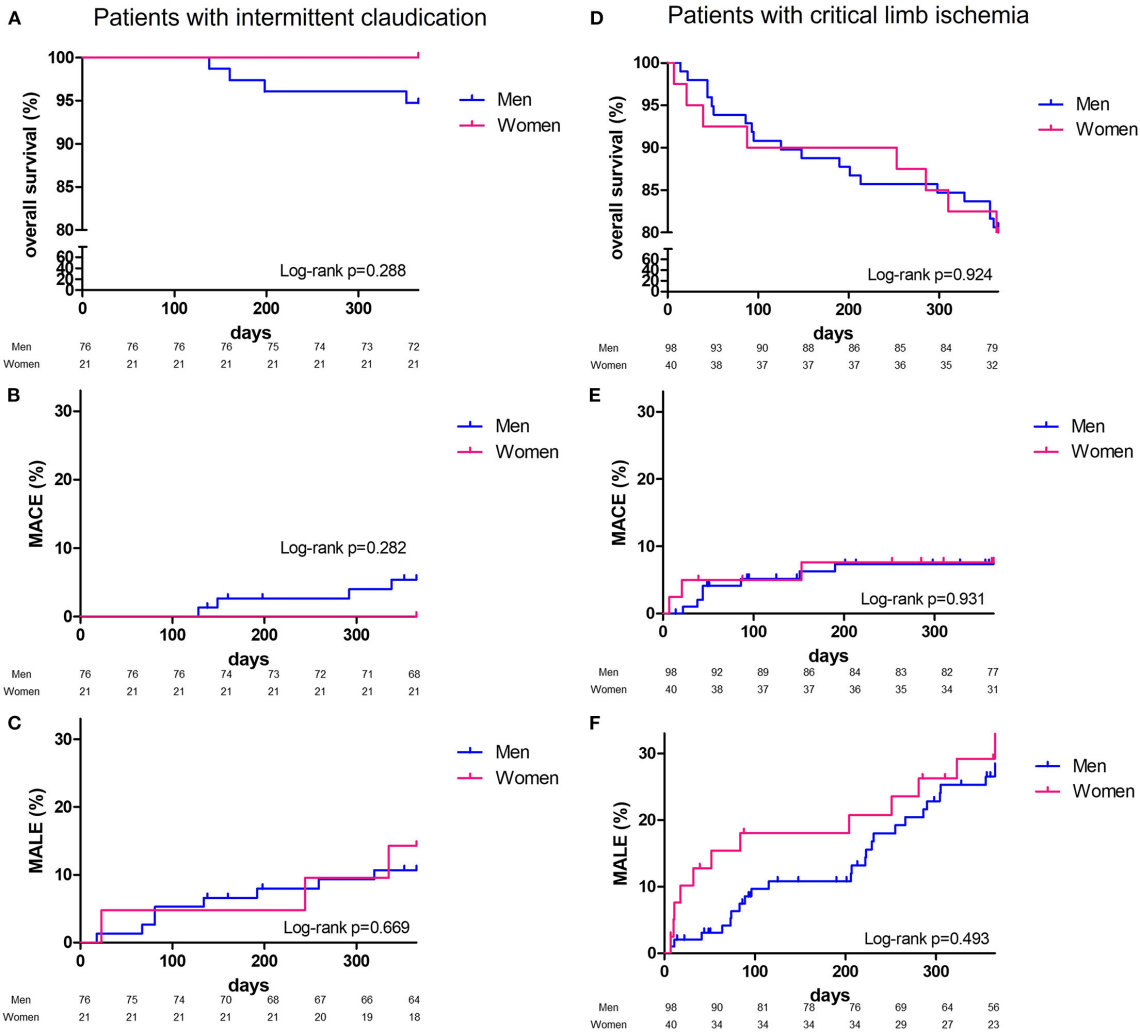
One-year Kaplan-Meier survival curves. Patients with intermittent claudicaion, overall survival **(A)**, major adverse cardiovascular events: MACE **(B)**, major adverse limb events: MALE **(C)**; patients with critical limb ischemia, overall survival **(D)**, major adverse cardiovascular events: MACE **(E)**, major adverse limb events: MALE **(F)** and comparison according to sex (men, blue line; women, red line) with Log Rank test comparisons between men (blue) and women (red).

At 1 year, overall survival, MACE or MALE did not differ between sex, with 23 (13.2%), 11 (6.3%), and 32 (18.4%) in men, and 8 (13.1%), 3 (4.9%), 15 (24.6%) in women, respectively (Table 2). Sepsis and cardiovascular events were the leading cause of death. Cancer annual incidence was 5%. Although non-statistically significant, MACE affected more men [11 (6.3%) men vs. 3 (4.9%) women], especially myocardial infarction, whereas it was the contrary for MALE affecting more women [15 (24.6%) men vs. 32 (18.4%) women], especially within the first 90 days (Figure 1). In patients with intermittent claudication (Rutherford category 1, 2, or 3) or critical limb ischemia (Rutherford category 4, 5, or 6) overall survival, MACE and MALE were similar between sex (Figure 2). Distribution of the level of revascularization was similar between men and women. Major amputation occurred in 3 (1.7%) men and 2 (3.3%) women, and minor amputations in 10 (5.7%) men and 3 (4.9%) women. When focusing on characteristics at inclusion on patients who died within 1 year, women were older than men with a median age of 87.6 (80.1–92.3) and 76.2 years (69.2–81.9) respectively *(p* = 0.02) and had more frequently a stroke in their past-medical history 3 (37.5%) vs. 1 (4.3%) for men (*p* = 0.04) (Table 3).

**Table 2:** One-year outcomes.

	**Total (*n* = 235)**	**Men (*n* = 174)**	**Women (*n* = 61)**	***p*-value**
Death	31 (13.2)	23 (13.2)	8 (13.1)	0.98
Median time to death - days (IQR)	148 (49–298))	148 (51–298)	171 (26–304)	0.75
Cause of death				0.63
Cardiovascular death	*7 (3.0)*	*5 (2.9)*	*2 (3.3)*	
Sepsis	*8 (3.4)*	*5 (2.9)*	*3 (4.9)*	
Cancer	*3 (1.3)*	*3 (1.7)*	*0 (0.0)*	
Hemorrhagic	*2 (0.9)*	*1 (0.6)*	*1 (1.6)*	
Unknown	*11 (4.7)*	*9 (5.2)*	*2 (3.3)*	
Cancer	11 (4.7)	8 (4.6)	3 (4.9)	1.00
MACE	14 (6.0)	11 (6.3)	3 (4.9)	1.00
Median time to MACE - days (IQR)	107 (34–163)	128 (44–190)	21 (14–87)	0.19
Myocardial infarction	10 (4.3)	10 (5.7)	*0 (0.0)*	0.07
Stroke	2 (0.9)	1 (0.6)	1 (1.6)	0.45
MALE	47 (20.0)	32 (18.4)	15 (24.6)	0.30
Median time to MALE - days (IQR)	192 (64–266)	199 (76–264)	84 (18–281)	0.40
Revascularization	45 (19.1)	31 (17.8)	14 (23.0)	0.38
Bypass procedures	*16 (6.8)*	*12 (6.9)*	*4 (6.6)*	
Aorto-iliac	*2 (0.9)*	*2 (1.1)*	*0 (0)*	
Femoro-popliteal	*11 (4.7)*	*7 (4.0)*	*4 (6.6)*	
Bellow the knee	*3 (1.3)*	*3 (1.7)*	*0 (0)*	
Endovascular procedures	*44 (18.7)*	*34 (19.5)*	*10 (16.4)*	
Aorto-iliac	*8 (3.4)*	*7 (4.0)*	*1 (1.6)*	
Femoro-popliteal	*26 (11.1)*	*19 (10.9)*	*7 (11.5)*	
Bellow the knee	*10 (4.3)*	*8 (4.6)*	*2 (3.3)*	
Major amputation	5 (2.1)	3 (1.7)	2 (3.3)	0.61
Minor amputation	13 (5.5)	10 (5.7)	3 (4.9)	1.00

*IQR, interquartile range (25th – 75th percentile); MACE, major adverse cardiovascular event; MALE, major adverse limb event. Figures in italic correspond to procedures and not patients*.

**Table 3:** Baseline characteristics of patients dead within 1 year and sex comparison.

	**Total (*n* = 31)**	**Men (*n* = 23)**	**Women (*n* = 8)**	***p*-value**
Median age at inclusion- years (IQR)	80.0 (69.5–86.1)	76.2 (69.2–81.9)	87.6 (80.1–92.3)	**0.02**
Past-medical history at inclusion				
Hypertension	24 (77.4)	18 (78.3)	6 (75.0)	1.00
Hyperlipemia	18 (58.1)	13 (56.5)	5 (62.5)	1.00
Smoking status				0.09
Never smoked	12 (38.7)	6 (26.1)	6 (75.0)	
Current smoker	6 (19.4)	6 (26.1)	0	
Former smoker	13 (41.9)	11 (47.8)	2 (25.0)	
Diabete mellitus	9 (61.3)	15 (65.2)	4 (50.0)	0.76
Stroke	4 (12.9)	1 (4.3)	3 (37.5)	**0.04**
Transient ischemic attack	0	0	0	0
Coronary artery disease	11 (35.5)	10 (43.5)	1 (12.5)	0.20
Myocardial infarction	8 (25.8)	7 (30.4)	1 (12.5)	0.64
Heart failure	5 (16.1)	4 (17.4)	1 (12.5)	1.00
Chronic obstructive pulmonary disease	4 (12.9)	4 (17.4)	0	0.55
Abdominal aortic aneurysm	2 (6.5)	2 (8.7)	0	1.00
Peripheral artery disease Rutherford category				0.27
Category 1/2/3 : Intermittent claudication	4 (12.9)	4 (17.4)	0	
Category 4: Pain while at rest	2 (6.5)	2 (8.7)	0	
Category 5/6 : Trophic lesion	25 (80.6)	17 (73.9)	8 (100)	
Previous bypass revascularization	7 (22.6)	5 (21.7)	2 (25.0)	1.00
Previous endovascular revascularization	13 (41.9)	11 (47.8)	2 (25.0)	0.41
Previous cardiovascular rehabilitation	**6 (19.4)**	3 (13.0)	3 (37.5)	0.16
Previous lower limb amputation	4 (12.9)	4 (17.3)	0	
Major amputation	1 (3.2)	1 (4.3)	0	1.00
Minor amputation	3 (9.7)	3 (13.0)	0	0.55
Revascularization before discharge	30 (96.8)	22 (95.7)	8 (100)	1.00

*IQR, interquartile range (25th – 75th percentile). Significant p values are in bold*.

## Discussion

In this study, we found that among patients hospitalized for PAD, women were older than men. However, there was no sex difference in overall mortality after 1 year of follow-up. Women smoked less and had less coronary artery disease, and we noticed a trend for fewer MACE and increased MALE vs. men. These data are consistent with the literature, where large studies observed that ASCVD outcomes were not higher in women compared with men (5).

Our study aimed at comparing characteristics and outcomes of patients hospitalized for PAD according to sex provides several key points. We did not find that women were under-treated or less likely to undergo revascularization than men (1, 6, 11, 12). Same rate of revascularization was observed in both men and women with even a trend to more procedures in women, but without any difference according to the level of limb revascularization (16, 17). Currently, the guidelines for symptomatic PAD recommend long-term treatments with at least antiplatelet agent and statin (7) to improve prognosis (18). Previous reports showed that this optimized therapy was less prescribed in women, suggesting a decrease in secondary prevention strategies in women compared to men (19). In our study, optimized therapy was approaching 80% of the patients whatever the sex. However, not all patients received statin at discharge. Forty patients (16.7%) patients either presented a previous intolerance or refused to take statin or ezetimibe. Our data are in accordance with large recent therapeutic trials. In the COMPASS trial (9), lipid-lowering therapy was present in 82.8 to 83.8% of the patients, and 73.0 to 73.7% in the EUCLID trial (8). No patient from this cohort had a PCSK9 inhibitor. This new lipid lowering class has been approved for reimbursement in France since 2020 only, when the LDL cholesterol goal is not achieved and in combination with a statin. The clinical presentation of the patients in this study was more severe than most of PAD cohorts (20–22) with almost 60% presenting with critical limb ischemia. Outpatients were not included in that study indeed, as a consequence, revascularizations for intermittent claudication were under-represented. Although intermittent claudication is considered a hallmark manifestation of PAD, women are more than twice as likely as men to report the presence of atypical exertional leg symptoms that sometimes began at rest or no symptoms at all (23, 24). Other associated comorbidities, such as osteoarthritis or osteoporosis, may also delay the diagnosis of PAD in women (25). Therefore, women have probably a long “latent phase” in which ASCVD and PAD progresses leading to more revascularization when hospitalized. This may explain why we observed that women presented with more tissue loss and less intermittent claudication as already reported (26). Interestingly, despite an increased age and the severe clinical presentation, the 1 year amputation-free survival was similar in both men and women (12, 27).

Women were older than men with almost 10 years apart when they presented with PAD in our study (28). One explanation may come from the fact that men were more current and former smokers than women, and would have develop PAD at younger age, as smoking, the most powerful contributor to PAD, increases the risk by 2 to 3 (29, 30). But another explanation may relate to the preponderant role that estrogens play in women, as reported decades ago in the Framingham study. Women in pre-menopause developed less coronary artery disease than women in post-menopause or women of same age with early menopause after ovariectomy indeed (31). Since, protective cardiovascular effects of estrogens have been demonstrated (32). They promote arterial vasodilation, decrease action of pro-inflammatory cytokines, lower low density lipoproteins and increase high density lipoproteins (32, 33). The drop in estrogen at menopause gives way to the development of ASCVD. But the impact of gonadotropins shall not be underestimated as secretion of follicle-stimulating hormone (FSH) from the pituitary gland begins to rise above normal levels before menopause, when estrogen levels are still normal, and become high after menopause responsible for a decrease of estrogen levels (34, 35). FSH levels were found to be correlated with the coronary calcium score and carotid intima-media thickness in women (36). Women with a lower increase in FSH during their transition to menopause may be less at risk of atherosclerosis than those with a medium or high increase in FSH at the same period (37, 38). Our team recently assessed the impact of gonadotropins *in vitro* on endothelial progenitor cells suggesting that gonadotropins blocking strategies could be a new interesting therapeutic approach in ASCVD (39).

Our study has some limitations. The limited number of patients included, and the short duration of follow-up make the power of this study insufficient to draw definitive conclusions. It may indeed underestimate the impact of sex on the characteristics, management, and prognosis of PAD patients. Also, our study was not powered for detecting differences in terms of hard cardiac or limb events between men and women. Our findings should be addressed with usual caution and would require studies within larger registries to be confirmed. International registries like the RECCORD registry (40), able to collect and sum-up data from multiple tertiary and non-tertiary centers would be very useful to provide a comprehensive dataset depicting the current real life practice and outcome of vascular care (40). We cannot provide details for intermittent claudication staging. We do not perform indeed a Strandness walking test for the hospitalized patients to provide these data. However, the patients requiring hospitalization in our tertiary center are severely impacted by their walking-distance and most report a walking distance below 200 m. However, the patients in our study shared the same cardiovascular risk factors and comorbidities than other PAD cohorts, where current smokers represented 16 to 39%, hypertension 63 to 81%, hyperlipidemia 57 to 67% and diabetes 26 to 44%. Besides PAD, patients had coronary artery disease in 24 to 52% and cerebrovascular disease in 13 to 23% (22, 41–45). Moreover, the accuracy of the data completion during follow-up was good as previously shown (14, 18, 41, 46).

## Conclusion

We showed that overall mortality, cardiovascular outcomes, limb revascularization or amputation did not differ between men and women, 1 year after hospitalization for PAD in our tertiary center. Although the latter were older, less smoker and had less coronary artery disease. Due to the small size of this cohort, larger studies and future research are needed to better understand sex-specific mechanisms in the pathophysiology and natural history of PAD, including sex hormones and gonadotrophins changes over adult life.

## Data Availability Statement

The original contributions presented in the study are included in the article, further inquiries can be directed to the corresponding author.

## Ethics Statement

The studies involving human participants were reviewed and approved by IRB CPP Sud Ouest et outre 79 mer II 07 021 08. The patients provided their written informed consent to participate in this study. Written informed consent was obtained from the individual(s) for the publication of any potentially identifiable images or data included in this article.

## Author Contributions

TM, EM, and DS: conception and design. HM and EM: administrative support. GD, CC, AGa, GG, LK, JS, MS, PJ, and TM: provision of study materials or patients. AGu, NM, OS, and HM: collection and assembly of data. AGu, GD, TM, DS, NG, and EM: data analysis and interpretation. All authors: manuscript writing and final approval of manuscript.

## Conflict of Interest

The authors declare that the research was conducted in the absence of any commercial or financial relationships that could be construed as a potential conflict of interest.

## Publisher's Note

All claims expressed in this article are solely those of the authors and do not necessarily represent those of their affiliated organizations, or those of the publisher, the editors and the reviewers. Any product that may be evaluated in this article, or claim that may be made by its manufacturer, is not guaranteed or endorsed by the publisher.
